# Double Perovskite Mn^4+^-Doped La_2_CaSnO_6_/La_2_MgSnO_6_ Phosphor for Near-Ultraviolet Light Excited W-LEDs and Plant Growth

**DOI:** 10.3390/molecules27227697

**Published:** 2022-11-09

**Authors:** Zheng Lu, Dashuai Sun, Zeyu Lyu, Sida Shen, Jianhui Wang, Hanwei Zhao, Lixuan Wang, Hongpeng You

**Affiliations:** 1 School of Rare Earths, University of Science and Technology of China, Hefei 230026, China; 2 Ganjiang Innovation Academy, Chinese Academy of Sciences, Ganzhou 341000, China; 3 State Key Laboratory of Rare Earth Resource Utilization, Changchun Institute of Applied Chemistry, Chinese Academy of Sciences, Changchun 130022, China

**Keywords:** red phosphor, luminescent properties, W-LEDs, plant growth

## Abstract

Non-rare earth doped oxide phosphors with far-red emission have become one of the hot spots of current research due to their low price and excellent physicochemical stability as the red component in white light-emitting diodes (W-LEDs) and plant growth. Herein, we report novel Mn^4+^-doped La_2_CaSnO_6_ and La_2_MgSnO_6_ phosphors by high-temperature solid-phase synthesis and analyzed their crystal structures by XRD and Rietveld refinement. Their excitation spectra consist of two distinct excitation bands with the dominant excitation range from 250 to 450 nm, indicating that they possess strong absorption of near-ultraviolet light. Their emission is located around 693 and 708 nm, respectively, and can be absorbed by the photosensitive pigments Pr and Pfr, proving their great potential for plant growth. Finally, the prepared samples were coated with 365 nm UV chips to fabricate far-red LEDs and W-LEDs with low correlation color temperature (CCT = 4958 K/5275 K) and high color rendering index (R_a_ = 96.4/96.6). Our results indicate that La_2_CaSnO_6_:Mn^4+^ and La_2_MgSnO_6_:Mn^4+^ red phosphors could be used as candidate materials for W-LED lighting and plant growth.

## 1. Introduction

As the latest generation of lighting sources, light-emitting diodes (LEDs) have been successfully used in many fields and have contributed to the development of these fields, especially in agriculture, because of their outstanding advantages such as high efficiency, environmental protection, and energy reduction [1,2,3,4,5]. As the most basic source of energy for the survival and reproduction of all living things on earth, the role played by light in the growth of plants is self-evident [6,7,8,9,10]. However, not all wavelengths of light are absorbed by the pigments in plants, with visible light (380–720 nm) and near-infrared light (720–800 nm) being the absorption ranges [11,12,13,14]. In contrast, near-ultraviolet (n-UV) light (290–380 nm) plays a negligible role in the plant growth process. In order to enable plants to utilize the energy of n-UV light, there is an urgent need for a light-converting phosphors that can convert n-UV light to visible or near-infrared light. Therefore, it is crucial to find phosphors that can be excited by n-UV light.

In recent years, Mn^4+^ ion has attracted much attention due to its abundant reserves and low prices. It is well known that Mn^4+^ ion is a good non-rare earth activator, which usually occupies an octahedral coordination environment in the lattice and has outstanding optical properties, including a broad absorption band covering the UV to visible region and characteristic red luminescence in the wavelength range from red to near-infrared light [15,16,17,18]. Intriguingly, the luminescence of the Mn^4+^ ions is right within the absorption range of the photosensitive pigments Pr (red light-absorbing form) and Pfr (far-red light-absorbing form) in plants. The photosensitive pigment is one of the most important photoreceptors for plant photomorphogenesis, and its role is indispensable for the developmental process of plants. It is not only involved in regulating plant growth and development, but also in responding to biotic and abiotic stresses. The absence of photosensitive pigments leads to altered plant resistance to biotic stresses such as pathogenic bacteria and pests, as well as abiotic stresses such as low temperature, high temperature, drought, and salt, while the resistance of plants to these adverse stresses can be improved by changing the light quality (e.g., adjusting the red far-red light ratio). Therefore, Mn^4+^-doped phosphors can be applied in plant growth.

There have been many reports on Mn^4+^-doped phosphors [5]. However, there have been no reports on the use of La_2_CaSnO_6_ and La_2_MgSnO_6_ crystals for the synthesis of red or deep-red luminescent phosphors. In this work, we report a series of novel red-emitting double perovskite structures of La_2_CaSnO_6_:Mn^4+^ (LCSO:Mn^4+^) and La_2_MgSnO_6_:Mn^4+^ (LMSO:Mn^4+^) phosphors obtained by high-temperature solid-phase reactions. The phase purity and crystal structures were characterized by XRD and Rietveld refinement. The luminescence properties of the prepared phosphors were investigated, including photoluminescence properties, temperature-dependent luminescence properties, quantum efficiency, and fluorescence lifetime. Furthermore, by combining with n-UV LED chips and commercial phosphors, both W-LEDs can produce white light with high CRI and low CCT. This paper demonstrates the potential of La_2_CaSnO_6_:Mn^4+^ and La_2_MgSnO_6_:Mn^4+^ phosphors for applications in W-LEDs and plant growth.

## 2. Results and Discussion

### 2.1. Structural Properties

**Figure 1:**
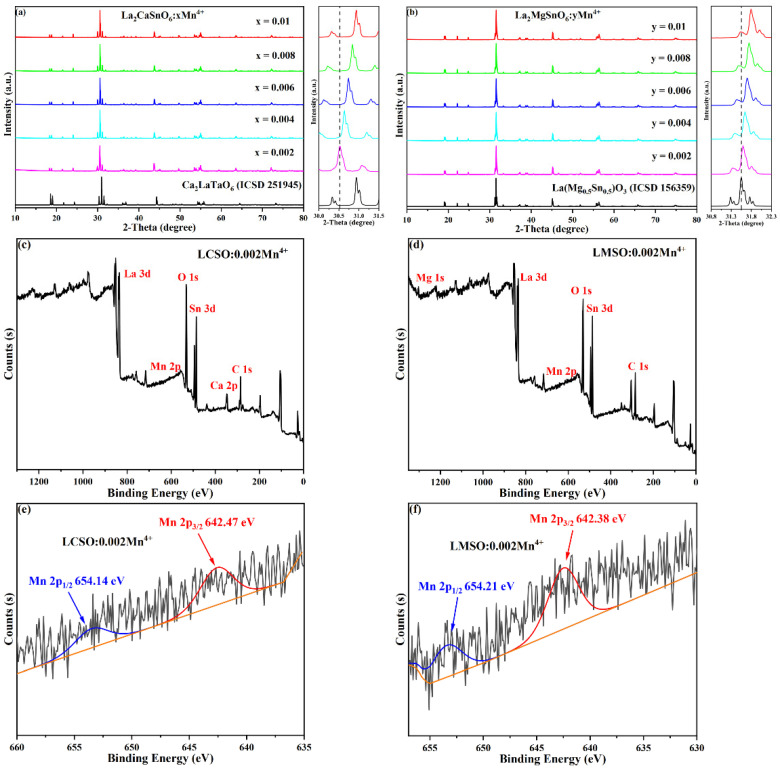
(**a**) XRD patterns of LCSO:xMn^4+^ (x = 0.002, 0.004, 0.006, 0.008, and 0.010). (**b**) XRD patterns of LMSO:yMn^4+^ (y = 0.002, 0.004, 0.006, 0.008, and 0.010). (**c**) XPS spectrum of the LCSO:0.002Mn^4+^. (**d**) XPS spectrum of the LMSO:0.002Mn^4+^. (**e**) Mn 2p XPS spectrum of the LCSO:0.002Mn^4+^. (**f**) Mn 2p XPS spectrum of the LMSO:0.002Mn^4+^.

Figure 1a,b show the XRD patterns of LCSO:xMn^4+^ (x = 0.002, 0.004, 0.006, 0.008, 0.010) and LMSO:yMn^4+^ (y = 0.002, 0.004, 0.006, 0.008, 0.010) samples. Obviously, the diffraction peaks of the Mn^4+^-doped samples with different concentrations match with those of the standard data and no impurity peaks are found, indicating that all the Mn^4+^ ions are dissolved in the main lattice. We tested the X-ray photoelectron spectra (XPS) of LCSO:0.002Mn^4+^ and LMSO:0.002Mn^4+^, and the results are shown in Figure 1c,d. The signals of La, Sn, Ca, Mg, and O elements were clearly detected. The XPS spectra of Mn 2p are shown in Figure 1e,f, and it can be observed that the binding energy peak of Mn 2P_1/2_ of both are located near 654 eV, and the binding energy peak of Mn 2p_3/2_ of both is located near 642 eV, which proves that the oxidation state of Mn is +4.

**Figure 2:**
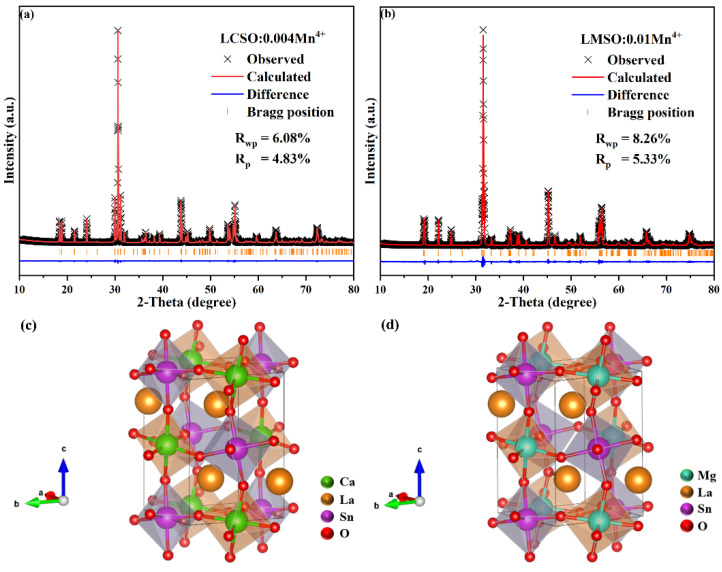
(**a**) Rietveld refinements of LCSO:0.004Mn^4+^. (**b**) Rietveld refinements of LMSO:0.01Mn^4+^. (**c**) Crystal structure of LCSO:0.004Mn^4+^. (**d**) Crystal structure of LMSO:0.01Mn^4+^.

To further explore the crystal structures of LCSO and LMSO, the crystal structure data of Ca_2_LaTaO_6_ (ICSD 251945) and La(Mg_0.5_Sn_0.5_)O_3_ (ICSD 156359) were used as starting base models for LCSO:0.004Mn^4+^ and LMSO:0.010Mn^4+^ Rietveld structure refinement, and the results are shown in Figure 2a,b [19,20]. The refinement results showed that LCSO:0.004Mn^4+^ and LMSO:0.010Mn^4+^ were pure phases. The detailed crystallographic data are shown in Table 1.

The crystal structures of LCSO and LMSO were obtained by refining the crystallization results in the *P*2_1_/*n* space group, and the results are shown in Figure 2c,d. La_2_CaSnO_6_ and La_2_MgSnO_6_ have the structural general formula A_2_BB’O_6_, where the A, B, and B’ sites are occupied by La, Ca/Mg, and Sn atoms, respectively. The B site binds to O^2−^ to form CaO_6_, MgO_6_, and SnO_6_ octahedra. The CaO_6_/MgO_6_ octahedron and the SnO_6_ octahedron have a common oxygen atom at the apex of both, and these two octahedra are staggered to form a long-range-ordered structural layer. The A site occupies the gap between the [BO_6_] octahedra and coordinates with twelve O^2−^. In the LCSO and LMSO crystal structures, there are three cations available for Mn^4+^ occupation. Considering the three aspects of ionic radius, valence state and coordination number, among the four ions La^3+^ ( r = 1.1 Å, CN = 7), Ca^2+^ ( r = 1 Å, CN = 6), Mg^2+^ ( r = 0.72 Å, CN = 6), and Sn^4+^ ( r = 0.69 Å, CN = 6), the Sn^4+^ site is considered the most suitable Mn^4+^ ( r = 0.53 Å, CN = 6) doping site.

### 2.2. Photoluminescence Properties

**Figure 3:**
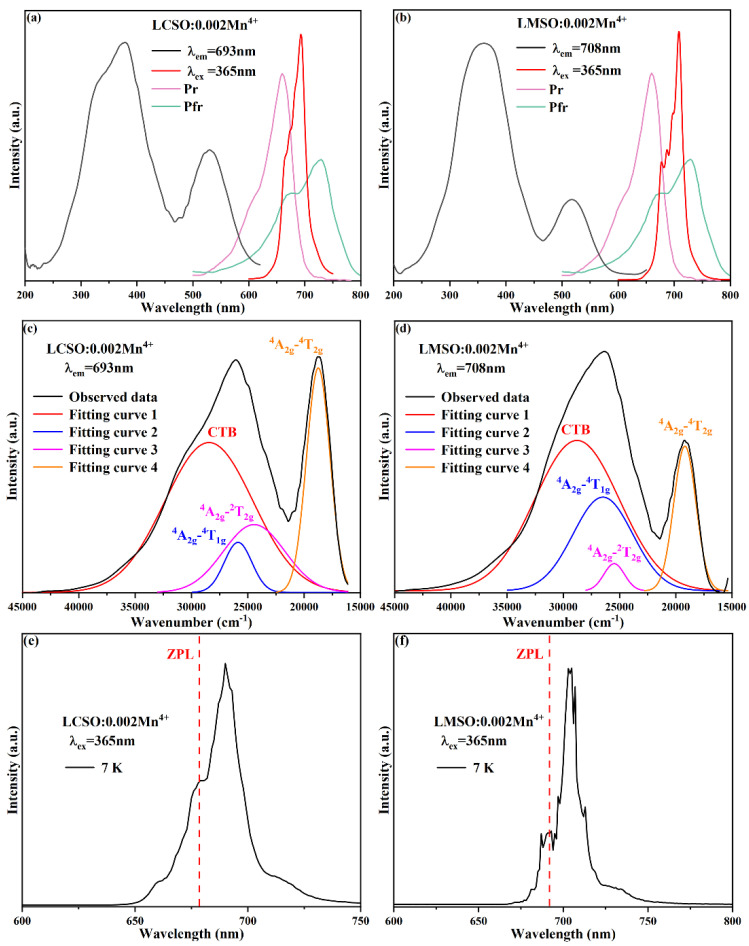
Excitation and emission spectra of (**a**) LCSO:0.002Mn^4+^ and (**b**) LMSO:0.002Mn^4+^. Gaussian fitting of the energy-based excitation spectra (**c**) LCSO:0.002Mn^4+^ and (**d**) LMSO:0.002Mn^4+^. Emission spectra of (**e**) LCSO:0.002Mn^4+^ and (**f**) LMSO:0.002Mn^4+^ at 7 K.

The excitation and emission spectra of La_2_CaSnO_6_:0.002Mn^4+^ and La_2_MgSnO_6_:0.002Mn^4+^ at room temperature are shown in Figure 3a,b. The photoluminescence excitation (PLE) spectra monitored at 693 and 708 nm show two distinct excitation bands with the dominant excitation range from 250 to 450 nm and the weak excitation range from 450 to 600 nm, due to the Mn^4+^-O^2−^ charge transition band (CTB), ^4^A_2g_ → ^4^T_1g_, ^4^A_2g_ → ^2^T_2g_, and ^4^A_2g_ → ^4^T_2g_ transitions [21,22]. As shown in Figure 3c,d, the Gaussian fit analysis of the energy-based PLE spectra of these two samples shows that the above four effects correspond roughly to 28,508 cm^−1^, 25,943 cm^−1^, 24,514 cm^−1^, and 18,768 cm^−1^ for LCSO:0.002Mn^4+^, and 28,816 cm^−1^, 26,381 cm^−1^, 25,458 cm^−1^, and 19,342 cm^−1^ for LMSO:0.002Mn^4+^. Both exhibit intense red emission under 365 nm UV excitation due to the spin-prohibited ^2^E_g_→^4^A_2g_ leap of the Mn^4+^ ions in the octahedral environment, with the peak emission near 693 nm for LCSO: 0.002Mn^4+^ and around 708 nm for LMSO: 0.002Mn^4+^, which can be absorbed by the photosensitive pigments Pr and Pfr [23]. These results prove that both phosphors are well suited for n-UV chips and are potential red-emitting phosphors, as well as able to be applied in plant growth. In addition, the emission spectra of both were found to show several sharp peaks. These peaks consist of the anti-Stokes emission, the zero-phonon line (ZPL) of the ^2^E_g_ → ^4^A_2g_ transition, and the Stokes emission [10,16]. Figure 3e,f show the emission spectra of LCSO:0.002Mn^4+^ and LMSO:0.002Mn^4+^ at 7 K. The ZPL of the ^2^E_g_ → ^4^A_2g_ transition was found to occur at 678 nm (14,749 cm^−1^) and 692 nm (14,451 cm^−1^), respectively.

**Figure 4:**
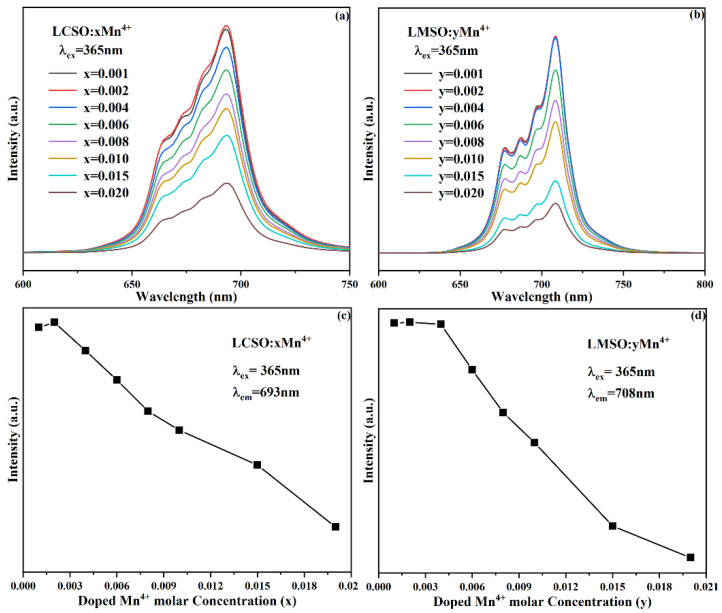
PL spectra of different amounts of Mn^4+^ in (**a**) LCSO and (**b**) LMSO under excitation at 365 nm. Relationship between doping concentration and emission intensity in (**c**) LCSO:xMn^4+^ and (**d**) LMSO:yMn^4+^.

**Figure 5:**
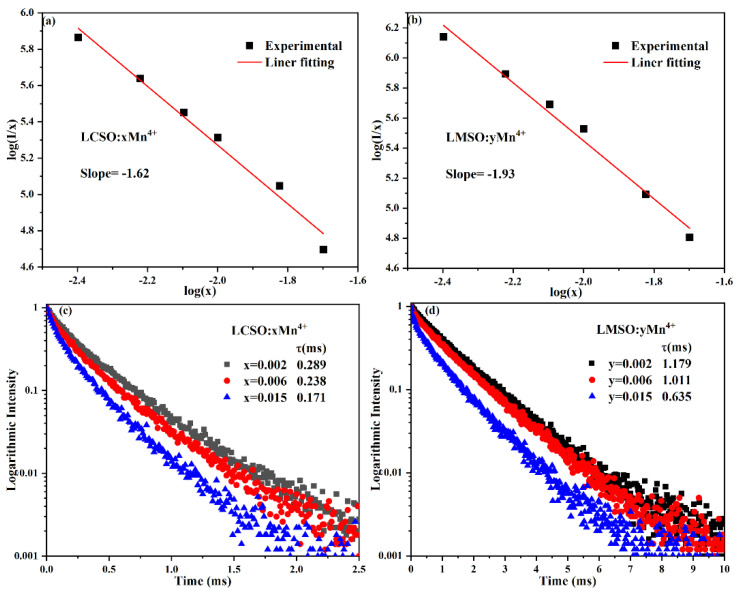
The linear fit of log(I/x) to log(x) of (**a**) LCSO:xMn^4+^ and (**b**) LMSO:yMn^4+^; fluorescence decay curves of (**c**) LCSO:xMn^4+^ and (**d**) LMSO:yMn^4+^.

The photoluminescence (PL) spectra of a series of different concentrations of LCSO:xMn^4+^ (x = 0.001–0.010) and LMSO:yMn^4+^ (x = 0.001–0.010) were tested as shown in Figure 4a,b. All samples with different doping concentrations show no absence and shift of emission peaks, only changes in emission intensity. From Figure 4c,d, it can be seen that LCSO:xMn^4+^ (x = 0.001–0.010) and LMSO:yMn^4+^ (y = 0.001–0.010) have the same trend, and the emission intensity increases with the increase in doping concentration from 0.001 to 0.002 and decreases with the increase from 0.002 to 0.01, and the optimal doping concentration for both was determined as 0.002. When the doping concentration exceeds 0.002, the emission intensity starts to decrease, and the concentration quenching appears and becomes more severe with the increase in the Mn^4+^ content. The concentration quenching includes two mechanisms: exchange interaction and multistage–multistage interaction. To investigate which mechanism causes the concentration quenching, we calculated the critical distance R_c_ by the following equation [24,25]:

(1) $$R_{c}\approx 2{\left(\frac{3V}{4{\pi X}_{c}N}\right)}^{\frac{1}{3}}$$

where V is the volume of the unit cell, x_c_ is the critical concentration, and N represents the number of sites that the Mn^4+^ ions can occupy per unit cell. For the LCSO host, V = 282.506 Å^3^, N = 2, and x_c_ = 0.002; the critical distance R_c_ is calculated to be 51.29 Å. For the LMSO host, V = 258.650 Å^3^, N = 2, and x_c_ = 0.002; the critical distance R_c_ is calculated to be 49.81 Å. Because the exchange interaction is effective only when the distance between activators is shorter than 5 Å, the concentration quenching mechanism of the Mn^4+^ ions in phosphors is dominated by multipole–multipole interactions. According to Van Uiter’s report, the emission intensity (I) per activator ion follows the equation [26]:

(2) $$\frac{I}{x}=K{\left[1+\beta {\left(x\right)}^{\frac{\theta }{3}}\right]}^{-1}$$

where x is the activator concentration, I/x is the emission intensity per activator concentration, K and β are the constants for the same excitation conditions, and θ = 6, 8, and 10 represent the dipole–dipole(d-d), dipole–quadrupole(d-q), and quadrupole–quadrupole(q-q) interactions. Figure 5a,b show a plot of log(I/x) versus log(x), with −θ/3 being the slope. For LCSO, a slope of −1.62 gives a θ of 4.86, which is closer to 6. For LMSO, a slope of −1.93 gives a θ of 5.79, again close to 6. These results suggest that dipole–dipole interactions dominate the concentration quenching mechanism of Mn^4+^ emission in LCSO and LMSO.

Figure 5c,d present luminescence decay curves of LCSO:xMn^4+^ (x = 0.002, 0.006, 0.015) and LMSO:yMn^4+^ (y = 0.002, 0.006, 0.015). All decay curves can be described by fitting the effective decay time τ, which is calculated as [27]:

(3) $$\tau =\frac{\int_{0}^{\infty }\mathrm{tI}\left(t\right)\mathrm{dt}}{\int_{0}^{\infty }I\left(t\right)\mathrm{dt}}$$

where I(t) means the luminescence intensity of Mn^4+^ at moment t. As the doping concentration of Mn^4+^ ions increases, the effective lifetimes were calculated to be 0.289, 0.238, and 0.171 ms for LCSO:xMn^4+^ (x = 0.002, 0.006, 0.015) and 1.179, 1.011, and 0.635 ms for LMSO:yMn^4+^ (y = 0.002, 0.006, 0.015), respectively. The decrease in the effective lifetimes is due to the increase in non-radiative energy transfer with the increase in Mn^4+^ doping concentration [28]. 

**Figure 6:**
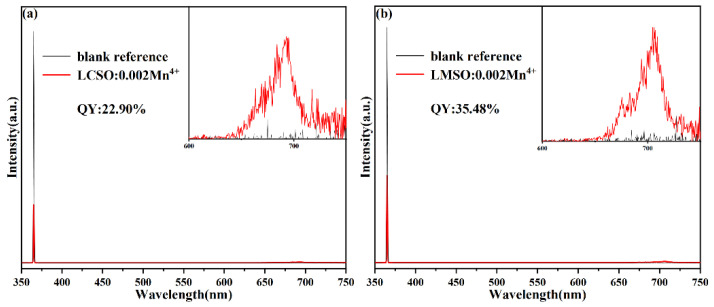
(**a**) IQY chart of LCSO:0.002Mn^4+^. (**b**) IQY chart of LMSO:0.002Mn^4+^.

In addition, we tested the internal quantum yields (IQYs) of LCSO:0.002Mn^4+^ and LMSO:0.002Mn^4+^, as shown in Figure 6. The IQYs were 22.90% for LCSO:0.002Mn^4+^ and 35.48% for LMSO:0.002Mn^4+^ under 365 nm excitation. The IQY of LMSO: 0.002Mn^4+^ is higher than some previously reported Mn^4+^-doped samples, as shown in Table 2.

### 2.3. Crystal Field Analysis and Nephelauxetic Effect

**Figure 7:**
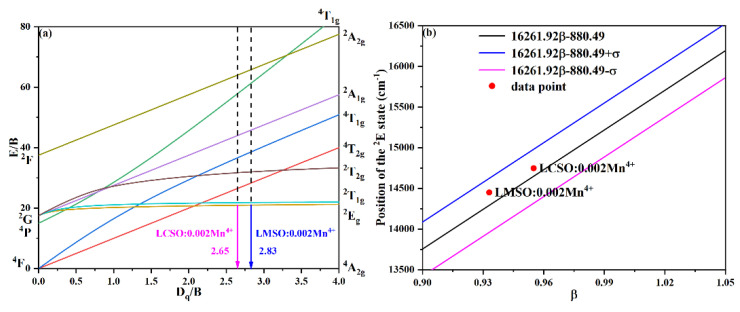
(**a**) Tanabe–Sugano diagram for Mn^4+^ (d^3^) electron configuration in the octahedral site of LCSO and LMSO host. (**b**) Dependence of energy of the Mn^4+ 2^E_g_ level on the nephelauxetic ratio.

In general, the Tanabe–Sugano energy level diagram can be used to explain the energy level splitting of the Mn^4+^ ions in a six-coordinate octahedral environment and its spectral properties, as shown in Figure 7a. When irradiated with an external light source, the electrons in the ground state ^4^A_2g_ absorb energy and jump to excited states ^4^T_1g_, ^4^T_2g_, and ^2^T_2g_, and then, through a non-radiative energy conversion process, to the lowest excited state ^2^E_g_, and finally return to the ground state; the energy is expressed in the form of red light. In this process, the crystal field strength (D_q_) strongly influences the energy level transition of the Mn^4+^ ions due to the unique 3d^3^ electron configuration of Mn^4+^ [32]. The crystal field strength is calculated from the energy difference of ^4^A_2g_ → ^4^T_2g_ by the following equation [33,34]:

(4) Dq=EA 42g−T 42g10

in addition, the Racah parameter B can be evaluated by the following equation [35]:

(5) $$\frac{{\;D}_{q}}{B}=\frac{15\left(x-8\right)}{x^{2}-10x}$$

the value of x can be calculated as follows [35]:

(6) x=EA 42g−T 41g−EA 42g−T 42gDq

the Racah parameter C can be acquired by the expression [35]:

(7) EE 2g−A 42gB=3.05CB+7.9−1.8BDq 

From the excitation spectrum and emission spectrum in Figure 3, the peak energies of the ^4^A_2g_ → ^4^T_1g_, ^4^A_2g_ → ^4^T_2g_, and ^2^E_g_ → ^4^A_2g_ are 25,943, 18,768, and 14,749 cm^−1^ for LCSO:0.002Mn^4+^ and 26,381, 19,342, and 14,451 cm^−1^ for LMSO:0.002Mn^4+^, respectively. Substituting the above data into Equation (4)–(7), the values of D_q_, B, C, and D_q_/B were calculated to be 1876.8 cm^−1^, 707.37 cm^−1^, 3160.88 cm^−1^, and 2.65 for LCSO:0.002Mn^4+^ and 1934.2 cm^−1^, 684.67 cm^−1^, 3107.66 cm^−1^, and 2.83 for LMSO:0.002Mn^4+^, respectively. The D_q_/B values of both are greater than two and prove that the Mn^4+^ ions possess strong crystal fields in LCSO and LMSO hosts. In addition, the ^2^E_g_ → ^4^A_2g_ transition of the Mn^4+^ ions is hardly affected by the crystal field strength as shown by the Tanabe–Sugano energy level diagram. What can affect such a transition is a nephelauxetic effect that depends on the covalent relationship between the Mn^4+^ ions and the ligand [23,36,37,38]. Brik et al. introduced the nephelauxetic ratio (β) to describe the nephelauxetic effect, which was calculated as follows [34]:

(8) $$\beta =\sqrt{{\left(\frac{B}{B_{0}}\right)}^{2}+{\left(\frac{C}{C_{0}}\right)}^{2}\;}$$

where B_o_ is 1160 cm^−1^ and C_o_ is 4303 cm^−1^ represent the Racah parameters of Mn^4+^-free ions. The β values of LCSO and LMSO were calculated as 0.955 and 0.933. On the basis of previous experience with a large number of Mn^4+^-doped crystals, a rule of thumb was established to predict the emission wavelength of Mn^4+^ with the following equation [32]:

(9) EE 2g−A 42g=16261.92β−880.49±σ

where σ is 332 cm^−1^, a value close to the typical energy of phonon modes [12,32]. As shown in Figure 7b, the ^2^E_g_ energy levels calculated by this empirical formula are in the range 14,318–14,982 cm^−1^ and 13,960–14,624 cm^−1^ for LCSO:0.002Mn^4+^ and LMSO:0.002Mn^4+^. The experimental values of the ^2^E_g_ → ^4^A_2g_ transition are 14,430 cm^−1^ for LCSO:0.002Mn^4+^ and 14,124 cm^−1^ for LMSO:0.002Mn^4+^, both of which are within the range.

### 2.4. Photoluminescence Thermal Stability

**Figure 8:**
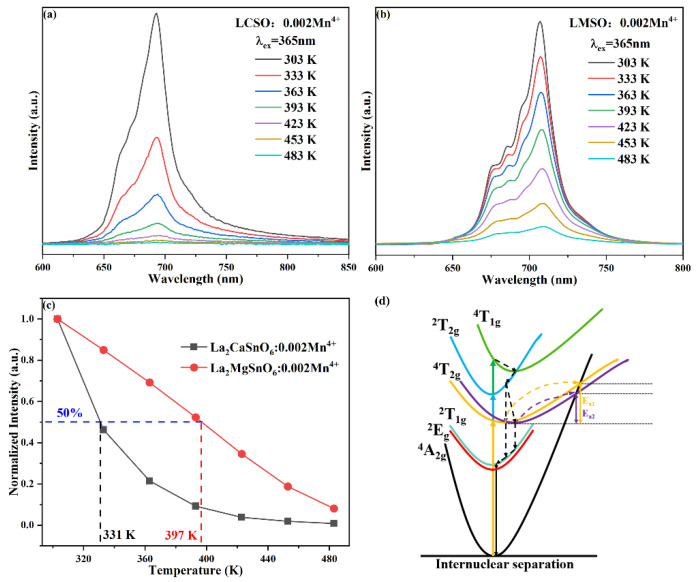
Emission intensity of (**a**) LCSO:0.002Mn^4+^ and (**b**) LMSO:0.002Mn^4+^ with temperature under 365 nm excitation. (**c**) Normalized intensity of the LCSO:0.002Mn^4+^ and LMSO:0.002Mn^4+^ as a function of temperature. (**d**) The configuration of the Mn^4+^ ions coordinate diagram.

**Figure 9:**
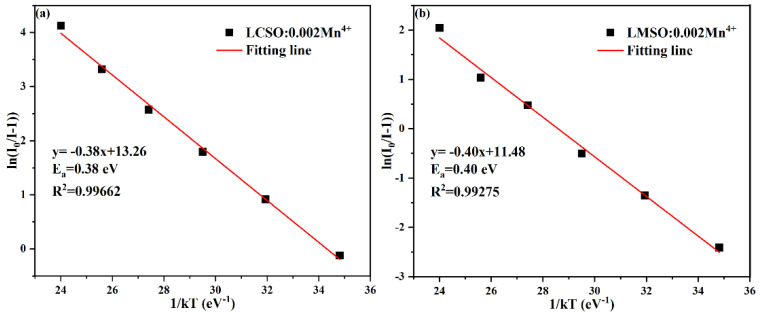
The linear relationship between ln(I_0_/I) and 1/kT and the calculated activation energy (E_a_) for LCSO: 0.002Mn^4+^ (**a**) and LMSO: 0.002Mn^4+^ (**b**).

Figure 8a,b show the emission spectra of LCSO:0.002Mn^4+^ and LMSO:0.002Mn^4+^ with temperature under 365 nm excitation. The increase in temperature leads to an increase in lattice vibrations, which increases the nonradiative transition and produces a thermal quenching effect with a significant decrease in the intensity of the emission peak [39]. The poor thermal stability of both is due to the significant Stokes shift of Mn^4+^ ions in the crystal environment. In addition, the normalized emission intensity curves of the two samples (Figure 8c) indicate that the quenching temperatures (T_50%_) of the LCSO:0.002Mn^4+^ and LMSO:0.002Mn^4+^ samples are about 331 and 397 K, respectively. The LCSO:0.002Mn^4+^ phosphor exhibits a more serious decay trend between the two. To better understand the thermal quenching properties, the activation energy (E_a_) is introduced to represent the energy difference between the cross-relaxation point and the excited state ^2^E_g_, which can be calculated by the Arrhenius formula [40]:

(10) $$\ln\left(\frac{I_{0}}{I}-1\right)=\mathrm{lnA}-\frac{E_{a}}{\mathrm{kT}}$$

where I_0_ is the initial emission intensity, I is the emission intensity at temperature T, A is a constant, E_a_ is the activation energy, and k is the Boltzmann constant equal to 8.626 × 10^−5^ eV/K. The specific linear relationship is described in Figure 9. E_a_ of LCSO:0.002Mn^4+^ is 0.38 eV, and E_a_ of LMSO:0.002Mn^4+^ is 0.40 eV. LCSO matrix structure is less stable than LMSO, resulting in lower activation energy and, thus, more prone to non-radiative processes, which eventually leads to a poor thermal stability. This situation is reflected in the configuration of the Mn^4+^ ions coordinate diagram (Figure 8d), indicating that smaller activation energy can excite the electron to reach the cross-relaxation point between the ground and excited states, followed by a radiation-free transition back to the ground state causing the quenching, thereby resulting in a lower quenching temperature [41].

### 2.5. Electroluminescence Spectrum of the Fabricated LED Devices

**Figure 10:**
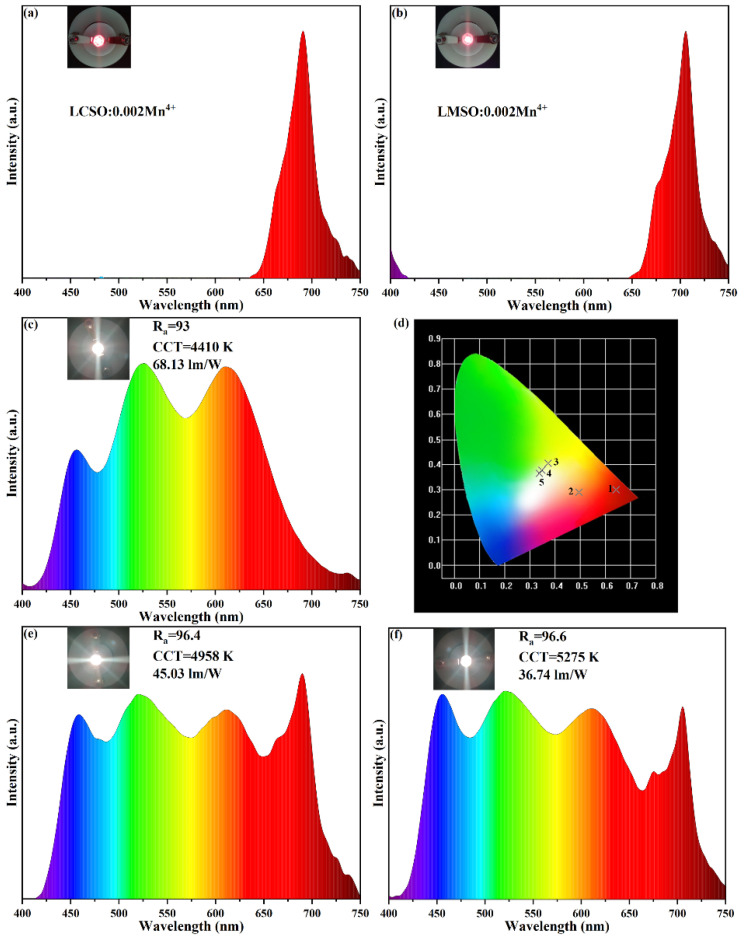
(**a**) Electroluminescence spectra of the LCSO:Mn^4+^. (**b**) Electroluminescence spectra of the LMSO:Mn^4+^. (**c**) Electroluminescence spectra of the BAM:Eu^2+^, (Sr,Ba)_2_SiO_4_:Eu^2+^, CaAlSiN_3_:Eu^2+^. (**d**) The CIE chromaticity coordinate graph. (**e**) Electroluminescence spectra of the BAM:Eu^2+^, (Sr,Ba)_2_SiO_4_:Eu^2+^, CaAlSiN_3_:Eu^2+^, and LCSO:Mn^4+^. (**f**) Electroluminescence spectra of the BAM:Eu^2+^, (Sr,Ba)_2_SiO_4_:Eu^2+^, CaAlSiN_3_:Eu^2+^, and LMSO:Mn^4+^.

To demonstrate the potential application of these two red phosphors in plant growth, we prepared two far-red LED models with electroluminescence spectra and luminescence photographs, as shown in Figure 10a,b. The CIE coordinates of (0.6431, 0.3006) and (0.4948, 0.2909) for both under a voltage of 3.36 V and a current of 20 mA are shown in Figure 10d (numeral 1 and numeral 2), respectively. Both far-red LED devices emitted bright far-red light under 365 nm excitation, and their emission spectra matched well with the absorption spectra of plant photosensitive pigments Pr and Pfr.

In addition, to demonstrate the potential application of these two red phosphors in W-LEDs, three W-LED models were prepared, and their electroluminescence spectra, luminescence photographs, and performance parameters (color rendering index, correlated color temperature, and lumen efficiency) are shown in Figure 10c,e,f. The CIE coordinates of (0.3716, 0.4052), (0.3486, 0.3770), and (0.3386, 0.3648) for the three with a voltage of 3.36 V and a current of 20 mA are shown in Figure 10d (numeral 3, numeral 4, and numeral 5), respectively. Comparing Figure 10e,f with Figure 10c, we found that the addition of these two phosphors was able to overcome the problem of missing far-red light, and the color rendering index increased from 93 to 96.4 and 96.6. The above results indicate that LCSO:0.002Mn^4+^ and LMSO:0.002Mn^4+^ red phosphors can be used as candidates for W-LEDs.

## 3. Materials and Methods

### 3.1. Sample Preparation

All La_2_CaSn_1−x_O_6_:xMn^4+^ (abbreviated as LCSO:xMn^4+^, x = 0–0.01) and La_2_MgSn_1-y_O_6_:yMn^4+^ (abbreviated as LMSO:yMn^4+^, y = 0–0.01) phosphors were synthesized by conventional high-temperature solid-state reaction. The raw materials were La_2_O_3_ (A.R.), CaCO_3_ (A.R.), MgO (A.R.), SnO_2_ (A.R.), and MnCO_3_ (5N), which were weighed in the appropriate chemical proportions. After 20 minutes of thorough grinding in an agate mortar, the resulting mixture was transferred to an alumina crucible, warmed up at 4 °C per minute, preheated at 900 °C for 2 hours, and then sintered at 1480 °C for 3 hours in an air atmosphere. Finally, the samples were naturally cooled to room temperature and then reground into homogeneous powder for further measurements.

### 3.2. Characterization

The phase purity of the sintered samples was analyzed using powder X-ray diffraction (XRD) (AXS D8 Advance, Bruker, Karlsruhe, Germany) with graphite monochromatic Cu Kα radiation (λ = 0.15405 nm) operating at 40 kV and 40 mA. Test from 10 to 80° in steps of 0.02° with a counting time of 0.14s per step. XRD Rietveld refinement was performed using the Structural Analysis System (GSAS) program (Created by Brian H. Toby, Argonne Laboratories, USA). The X-ray photoelectron spectroscopy (XPS) data were obtained through a Thermo Scientific K-Alpha instrument (Thermo Fisher Scientific, Waltham, Massachusetts, USA) with a monochromatized Al Kα line source, and the binding energy was calibrated to the C 1s peak at 284.8 eV. Photoluminescence emission (PL) and excitation (PLE) spectra were measured on a fluorescence spectrophotometer (F-7100, Hitachi, Tokyo, Japan) equipped with a 150 W xenon lamp. The luminescence decay curves and quantum yields (QY) were measured by an Edinburgh Instruments FLS-980 spectrometer (Edinburgh Instruments, Edinburgh, UK) equipped with an Edinburgh Instruments integrating sphere. The temperature-dependent photoluminescence emission (PL) spectra were tested on a phosphor excitation spectrum with thermal quenching analysis system EX-1000 (Hangzhou, China). The commercial blue phosphor BAM:Eu^2+^, green phosphor (Sr,Ba)_2_SiO_4_:Eu^2+^, red phosphor CaAlSiN_3_:Eu^2+^, the as-synthesized La_2_(Ca,Mg)SnO_6_:0.002Mn^4+^, and UV-LED chips (365 nm) were used to fabricate W-LEDs. The luminescence characteristics of the white LED were evaluated using a Starspec SSP6612 (forward bias current, 20 mA, forward voltage, 3.36V) (Hangzhou, China).

## 4. Conclusions

In summary, a series of La_2_(Ca/Mg)SnO_6_:Mn^4+^ red phosphors were successfully prepared using a conventional high-temperature solid-state reaction. The Rietveld refinements revealed that both La_2_CaSnO_6_:Mn^4+^ and La_2_MgSnO_6_:Mn^4+^ were monoclinic phases and the Mn^4+^ ions replace the Sn^4+^ ions. The Tanabe–Sugano energy level diagrams indicate that the Mn^4+^ ions were in a strong crystal field. In addition, LCSO:0.002Mn^4+^ and LCSO:0.002Mn^4+^ quantum yields were 22.90% and 35.48%, respectively. The thermal quenching phenomena of LCSO:0.002Mn^4+^ and LMSO:0.002Mn^4+^ were explained by the conformational coordinate diagram of the Mn^4+^ ions. Finally, the obtained far-red LED devices emitted bright far-red light that matched well with the absorption spectra of plant photosensitive pigments Pr and Pfr. Additionally, the prepared W-LEDs had R_a_ of 96.4 and 96.6 and CCT of 4958 K and 5275 K, respectively. These results show that LCSO:0.002Mn^4+^ and LMSO:0.002Mn^4+^ red phosphors can be used as the red component of W-LEDs as well as in plant growth.

## Figures and Tables

**Table 1:** Main crystallographic parameters of LCSO:0.004Mn^4+^ and LMSO:0.010Mn^4+^.

Compound	La_2_CaSnO_6_:0.004Mn^4+^	La_2_MgSnO_6_:0.010Mn^4+^
Space group	*P*2_1_/*n*	*P*2_1_/*n*
a, Å	5.74019	5.63776
b, Å	5.95969	5.72036
c, Å	8.25804	8.02015
α = γ	90°	90°
β	89.99°	90.06°
V	282.506	258.650
Crystal system	Monoclinic	Monoclinic
Z	2	2
R_wp_, %	6.08	8.26
R_p_, %	4.83	5.33
χ^2^	1.008	2.173

**Table 2:** The luminescence properties of La_2_(Ca/Mg)SnO_6_:Mn^4+^ and other Mn^4+^-doped phosphors.

Phosphor Composition	λ_ex_ (nm)	λ_em_ (nm)	IQY	References
Ba_2_YNbO_6_:Mn^4+^	360	695	29.2	[17]
Ca_2_YSbO_6_:Mn^4+^	470	680	19.72	[18]
SrLaGa_3_O_7_:Mn^4+^	380	715	13.83	[21]
La_2_MgTiO_6_:Mn^4+^	350	710	32.5	[29]
Li_2_MgTiO_4_:Mn^4+^	460	676	32	[30]
Li_2_MgZrO_4_:Mn^4+^	335	670	32.3	[31]
LCSO:Mn^4+^	365	693	22.90	This work
LMSO:Mn^4+^	365	708	35.48	This work
